# Distraction-Related Phenomena in Emergency Department Work: A Systematic Literature Review from a Cognitive Ergonomics Perspective

**DOI:** 10.3390/jcm15218258

**Published:** 2026-10-23

**Authors:** Petra Klanjšek, Matej Marin, Barbara Kegl, Jernej Mori, Zvonka Fekonja

**Affiliations:** 1 Faculty of Health Sciences, University of Maribor, Žitna Ulica 15, 2000 Maribor, Slovenia; petra.klanjsek@um.si (P.K.); matej.marin@student.um.si (M.M.); barbara.kegl@um.si (B.K.); 2 Emergency Department, University Medical Centre Maribor, Ljubljanska Ulica 5, 2000 Maribor, Slovenia; jernej.mori@ukc-mb.si

**Keywords:** workflow interruptions, mental workload, cognitive ergonomics, human factors, emergency department, patient safety

## Abstract

**Background**: Emergency departments are cognitively demanding environments where healthcare professionals are frequently exposed to interruptions and other distracting factors that may compromise workflow and patient safety. This systematic review aimed to identify factors that distract healthcare professionals in emergency departments and to synthesise their cognitive, organisational, and patient-safety consequences from a cognitive ergonomics perspective. **Methods**: A systematic literature review was conducted in PubMed, CINAHL Ultimate, Web of Science, and ScienceDirect in accordance with the PRISMA 2020 statement. Studies meeting predefined eligibility criteria were included. Methodological quality was assessed using design-specific critical appraisal tools, and findings were synthesised using inductive content analysis. **Results**: Nineteen studies met the predefined eligibility criteria and were included in the synthesis. The most frequently reported distraction-related phenomena included communication-related interruptions, telephone calls, and technological and environmental disturbances, while multitasking and task switching were identified as related work processes or responses to competing demands. Across the studies, distraction-related phenomena were associated with cognitive and workflow consequences, including increased workload, reduced situation awareness, work-related stress, and workflow disruption. Only one of the 19 included studies directly examined task errors, whereas the others identified interruptions during medication-related activities and potential safety concerns without directly measuring medication errors or patient harm. Several studies also identified clinically relevant interruptions associated with information exchange and care coordination, alongside unnecessary or poorly timed interruptions that were perceived as disruptive. **Conclusions**: Distraction-related phenomena are common features of emergency department work and are associated with cognitive and workflow outcomes. However, direct evidence regarding clinical errors and patient safety outcomes remains limited, and the available findings do not establish a causal relationship between distraction-related phenomena and patient harm. Potential cognitive-ergonomic strategies may focus on reducing avoidable and low-value interruptions while preserving clinically necessary communication that supports information exchange and care coordination; however, their effectiveness requires empirical evaluation.

## 1. Introduction

Emergency departments (EDs) are highly dynamic and cognitively demanding healthcare environments characterised by time pressure, clinical uncertainty, frequent communication, and competing task demands [1,2,3]. Healthcare professionals working in these settings must continuously prioritise tasks, manage interruptions, and make rapid decisions, often under conditions that may compromise attention, workflow continuity, and patient safety [3,4,5].

In this review, distraction factors are defined as environmental, organisational, technological, and communication-related stimuli or events that divert healthcare professionals’ attention from a primary task during emergency care delivery. This umbrella concept encompasses interruptions, workflow disruptions, environmental noise, monitor alarms, competing clinical demands, and other events that require healthcare [3,4,5] professionals to switch tasks or redirect their attention. Within this framework, interruptions are discrete events that intrude on or suspend an ongoing task, whereas workflow disruptions are broader disturbances that affect task coordination, continuity, and the overall functioning of the clinical work system. Noise and alarm exposure are recognised as environmental distraction factors, whereas multitasking and task switching are conceptualised as work processes through which competing demands are managed. Mental workload, in contrast, is conceptualised primarily as a cognitive response to the demands imposed by the work system rather than as a distraction factor itself [6].

Given the heterogeneous and partly overlapping terminology used in the literature, interruptions and distraction factors are not treated as synonymous in this review. Instead, interruptions represent one form of distraction-related phenomenon within the broader conceptual framework adopted for this review. This distinction allows distraction-related phenomena to be examined beyond interruptions alone and within the broader interplay of environmental, organisational, technological, and communication-related demands in the emergency department work system [7,8]. 

Examining distraction factors through the lens of cognitive ergonomics is particularly important because this perspective focuses on how the characteristics of the work system shape attention allocation, task switching, working memory, situation awareness, and clinical decision-making in real time [8,9]. Whereas patient safety frameworks primarily focus on adverse events and occupational stress frameworks emphasise workload and staff well-being, cognitive ergonomics offers a mechanistic explanation of how distractions, interruptions, multitasking, communication demands, and environmental conditions influence cognitive processes and ultimately affect clinical performance in emergency care [8,10].

EDs are highly complex work systems characterised by high patient volumes, clinical complexity, time pressure, and competing task demands [2,11,12]. These conditions contribute to elevated mental workload and stress, which may affect attention, situational awareness, and clinical performance [3,13,14,15]. From a cognitive ergonomics perspective, interruptions, noise, communication demands, and technology-related disturbances interact with these broader work-system demands and may further increase cognitive burden and compromise task performance and patient safety [1,4,5,16].

Previous research has provided important insights into interruptions in emergency care. Danesh [7] systematically reviewed interruptions in the emergency department work environment, with a primary focus on the characteristics, sources, and consequences of interruption events. Werner and Holden [17], in turn, proposed a sociotechnical model illustrating how interruptions arise from interactions among people, tasks, technologies, organisational structures, and the physical environment. Building on these contributions, the present review adopts a broader cognitive ergonomics perspective by distinguishing interruptions from related but conceptually different phenomena, including workflow disruptions, multitasking, and task switching, while treating mental workload as a cognitive response rather than as a distraction factor. It further synthesises how these distraction-related phenomena are associated with cognitive processes, workflow and task performance, emotional responses, patient-related outcomes, and context-dependent effects, including the distinction between disruptive and clinically functional interruptions. Thus, rather than reviewing interruption frequency and sources alone, the present review examines how distraction-related phenomena interact with cognitive demands and the wider work system in emergency care.

Environmental noise represents an additional source of distraction in emergency care. Noise levels in emergency settings frequently exceed recommended limits and have been associated with impaired communication, increased mental workload, reduced staff well-being, and potential threats to patient safety [18,19,20,21]. This further supports the examination of distraction-related phenomena as interacting components of the emergency department work system rather than as isolated environmental or organisational stressors.

Therefore, this systematic literature review aimed to extend previous interruption-focused research by identifying, categorising, and synthesising a broader range of distraction-related phenomena affecting healthcare professionals in emergency departments, and to examine their cognitive, organisational, and patient-safety consequences from a cognitive ergonomics perspective. More specifically, the review sought to: (1) identify the main types and sources of distraction factors in emergency departments; (2) examine their effects on workflow, attention, mental workload, situation awareness, and decision-making; (3) distinguish between harmful distractions and functional or clinically justified interruptions; and (4) derive implications for work-process design, communication practices, and interruption-management strategies in emergency care.

**Figure 1:**
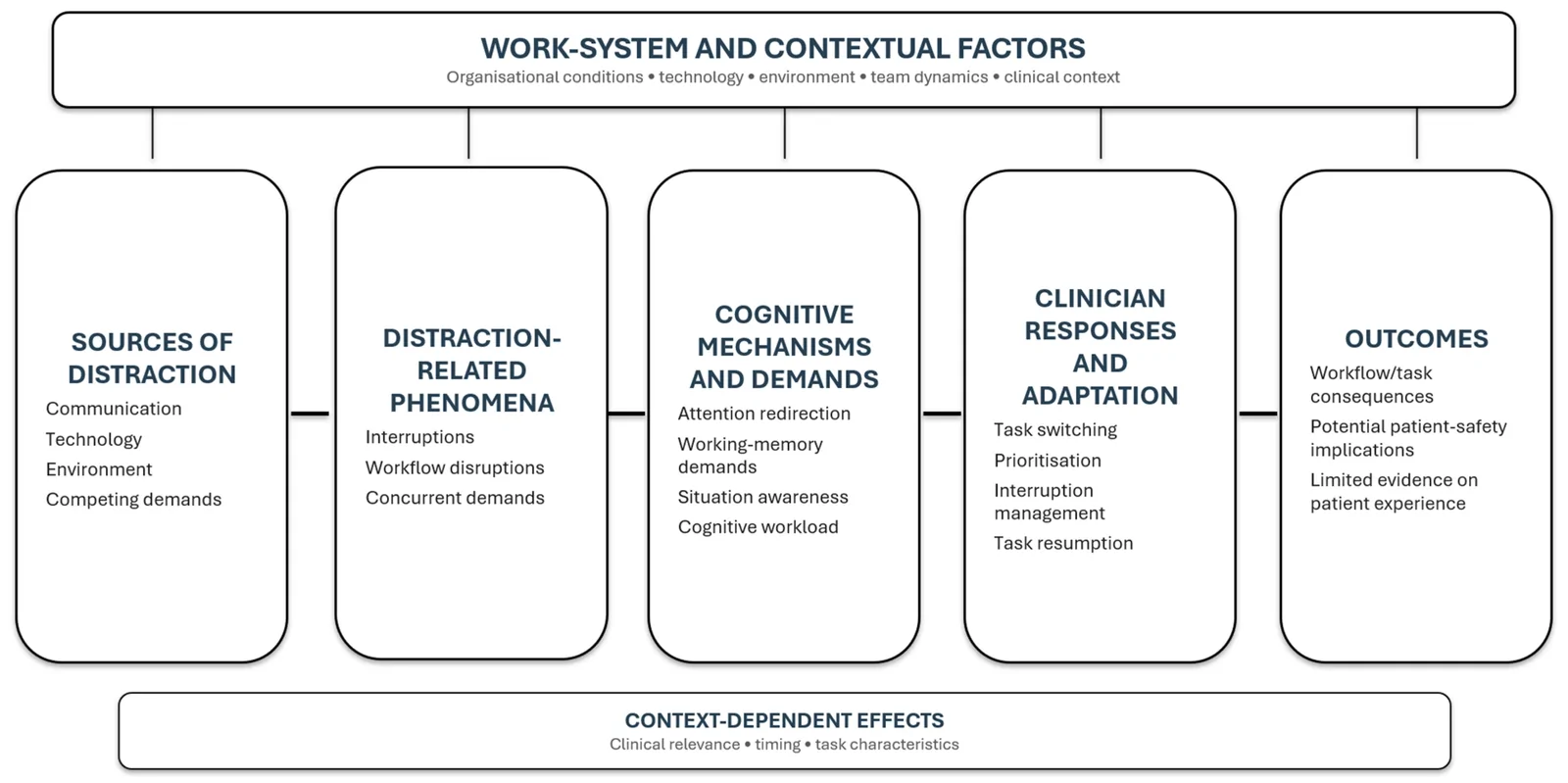
Conceptual cognitive-ergonomic framework for interpreting distraction-related phenomena in emergency department work.

Cognitive ergonomics serves as the primary theoretical framework for this review, providing the basis for understanding how distraction-related phenomena interact with attention, working memory, situation awareness, task switching, and clinical performance. Sociotechnical systems thinking, organisational accident theory, and Safety-I/Safety-II perspectives provide complementary explanatory perspectives by extending this interpretation to interactions among clinicians, tasks, technologies, organisational conditions, and adaptive work practices. These perspectives are therefore used as complementary levels of explanation rather than as separate or competing theoretical frameworks. The conceptual relationships guiding the interpretation of the review findings are summarised in Figure 1.

## 2. Materials and Methods

### 2.1. Study Design

This study was conducted as a systematic literature review to identify, critically appraise, and synthesise evidence on distraction-related phenomena affecting healthcare professionals in emergency departments (EDs), with particular attention to their cognitive, organisational, workflow-related, emotional, and patient-related consequences. The review further examined the context-dependent nature of interruptions, including their potentially disruptive as well as functional roles in emergency care. A systematic review methodology was selected to enable the comprehensive identification, appraisal, and synthesis of heterogeneous evidence concerning distraction-related phenomena in emergency care.

The review was reported in accordance with the PRISMA 2020 statement [22]. The PRISMA 2020 checklist (Supplementary File S1) was used to guide the reporting of the review process, including eligibility criteria, information sources, search strategy, study selection, data extraction, critical appraisal, and synthesis of findings. The study selection procedure is illustrated in the PRISMA 2020 flow diagram. The review was not prospectively registered with PROSPERO or another review registry, and no standalone formal review protocol was prepared. The research question, eligibility criteria, information sources, and general methodological approach were established during the initial planning phase of the review. The study selection and data-extraction procedures, critical appraisal methods, and synthesis approach are described in detail in the following sections. A detailed account of the review methodology, including the eligibility criteria, study selection and data extraction procedures, critical appraisal methods, planned synthesis approach, and the distinction between initial methodological decisions and analytical elements developed during the synthesis, is provided in Supplementary File S2.

The synthesis was conducted using inductive content analysis. Accordingly, the specific codes, subcategories, and analytical categories were developed and refined through iterative examination and comparison of the findings from the included studies. Although cognitive ergonomics provided the overarching theoretical perspective for the review, it was not used as an a priori coding framework. Instead, the categories were derived inductively from the included studies and subsequently interpreted through a cognitive ergonomics lens to examine the relationships between distraction-related phenomena, cognitive demands, workflow characteristics, and patient-safety implications. These analytical developments were therefore distinguished from the methodological procedures used to identify, select, and appraise the included studies.

The research question was developed according to the PIO (Population, Interest, Outcome) framework [23], with healthcare professionals working in emergency departments representing the population (P), distraction-related phenomena representing the phenomenon of interest (I), and their cognitive, workflow-related, emotional, and patient-related consequences representing the outcomes (O). Organisational and sociotechnical factors, as well as the context-dependent effects of interruptions, were also considered to capture the broader cognitive-ergonomic context of emergency department work. Accordingly, the following research question was formulated: “What distraction-related phenomena affect healthcare professionals in emergency departments, what consequences do they have, and under which organisational and sociotechnical conditions may interruptions be disruptive or beneficial?”

### 2.2. Search Strategy

A systematic literature search was conducted in PubMed, CINAHL Ultimate, Web of Science, and ScienceDirect (Elsevier). To complement the database searches, a supplementary search was conducted in Google Scholar using the same search string as in PubMed, CINAHL Ultimate, and Web of Science (Supplementary Table S1). Search results were screened using Google Scholar’s relevance-based ranking, with screening limited to the first 5 pages (50 records). The dynamic nature of Google Scholar’s relevance-based ranking and the limited depth of screening limit the reproducibility and comprehensiveness of this approach. Backward citation searching was performed by manually examining the reference lists of all included studies and relevant review papers to identify additional potentially eligible primary studies. Forward citation searching was not conducted. The final search was conducted on 15 March 2026. No restriction regarding publication year was applied; however, only studies published in English were considered eligible. 

The search strategy was developed using combinations of free-text keywords. Search terms related to the emergency department setting, distraction-related phenomena, and healthcare professionals were combined using Boolean operators (AND, OR). The search syntax was adapted to each database’s indexing system and technical requirements while maintaining conceptual equivalence across databases.

The search terms were developed from the predefined PIO framework and the review’s conceptual domains, with terms representing the emergency department setting, distraction- and interruption-related phenomena, relevant outcomes, and healthcare professionals. Because terminology concerning distraction-related phenomena is heterogeneous across the literature, free-text terms and synonymous expressions were combined and adapted to the technical requirements of each database. The complete database-specific search strategies, including the exact search strings used in each database, are provided in Supplementary Table S1.

The primary search strategy was as follows: (“ER” OR “emergency room” OR “emergency department” OR “ED”) AND (distraction* OR “distracting event*” OR noise* OR interference* OR disturb* OR interruption* OR “work interruption*” OR “cognitive load*” OR “alarm*”) AND (“stress level*” OR “patient care” OR “mental stress”) AND (“healthcare worker*” OR nurse* OR “medical staff”). 

Due to limitations in the use of Boolean operators and keyword combinations in the ScienceDirect database, a simplified search string was applied: (“emergency department” OR “emergency room”) AND (distraction* OR noise* OR “work interruption*”) AND (“healthcare worker*” OR nurse*) AND (stress OR “patient safety”).

### 2.3. Inclusion and Exclusion Criteria

For study selection and synthesis, distraction-related phenomena were operationalised as events, stimuli, or competing demands that diverted attention from, interrupted, or interfered with an ongoing clinical task or workflow. This included interruptions, environmental and technology-related distractions, competing clinical demands, and related workflow disruptions. Multitasking and task switching were considered related work processes through which competing demands were managed, whereas mental workload was treated primarily as a cognitive response or consequence rather than as a distraction factor itself. Given the heterogeneous terminology used across the literature, studies were considered eligible when the phenomena investigated were conceptually consistent with this operational framework, irrespective of the terminology used by the original authors.

Mental workload, stress, emotional responses, situation awareness, and task-performance outcomes were classified as consequences when they were examined as responses to distraction-related phenomena or competing work demands. Studies examining these outcomes without an identifiable distraction-related phenomenon, interruption, competing demand, or related work process were not considered eligible. In ambiguous cases, eligibility was determined by examining the study aim, operational definitions, exposure–outcome relationship, and reported analytical framework, with disagreements resolved through reviewer discussion and consensus.

Studies were included if they investigated distraction factors affecting the work of healthcare professionals in emergency departments. Eligible studies were full-text primary research articles published in peer-reviewed scientific journals using quantitative, qualitative, and mixed-methods research. Reviews were not included as units of analysis but were used for supplementary reference-list screening to identify potentially eligible primary studies. Only studies published in English were considered eligible. Grey literature, dissertations and theses, conference abstracts and proceedings, preprints, and unpublished studies were not systematically searched and were not eligible for inclusion (Table 1).

Studies were excluded if they did not address the review question, were not conducted in an emergency department, did not focus on healthcare professionals, or did not investigate distraction-related factors. Duplicate records, conference abstracts, opinion papers, commentaries, and publications that did not meet the inclusion criteria were also removed.

### 2.4. Selection and Data Extraction

The study selection process was conducted in accordance with the PRISMA 2020 statement [22]. Three reviewers (M.M., P.K. and J.M.) independently screened the titles and abstracts of all retrieved records against the predefined eligibility criteria.

Potentially eligible articles were subsequently assessed in full text by the same three reviewers. 

Disagreements at either stage were resolved through discussion and consensus among the three screening reviewers. When consensus could not be reached, two additional reviewers (B.K. and Z.F.) were consulted. Reasons for exclusion at the full-text stage were documented. Inter-rater agreement was not formally quantified.

Following completion of the study-selection process, data were independently extracted from the included studies by two reviewers using a standardised data extraction form. The data-extraction framework, including the predefined variables extracted from the included studies, is presented in Supplementary Table S2. Both reviewers independently extracted the complete predefined dataset from each study, thereby providing independent verification of all extracted data rather than verification limited to discrepancies. The extracted datasets were subsequently compared, and any discrepancies were resolved through discussion and consensus. The data-extraction form was not formally piloted before use. Extracted information included the author(s), year of publication, country, study design, study setting, participant characteristics, sample size, distraction factors investigated, outcome measures, and the main findings relevant to the review question.

### 2.5. Critical Appraisal of Studies

The methodological quality of the included studies was assessed using design-specific critical appraisal tools [24,25,26]. Quantitative observational studies with a cross-sectional or descriptive observational structure were appraised using the JBI Checklist for Analytical Cross-Sectional Studies [24], while the prospective cohort study was assessed using the JBI Checklist for Cohort Studies [25]. Qualitative studies were evaluated using the JBI Checklist for Qualitative Research [26], and mixed-methods studies were assessed using the Mixed Methods Appraisal Tool (MMAT), version 2018 [27]. Each study was independently evaluated by two reviewers (M.M. and P.K.). Discrepancies in the assessment were resolved through discussion and consensus, with additional reviewers (B.K. and Z.F.) involved when necessary. Appraisal findings were reported at the level of individual methodological domains using the response categories “yes,” “no,” “unclear,” and “not applicable,” in accordance with the respective appraisal tools. No numerical summary scores or thresholds were used to classify studies according to methodological quality.

No studies were excluded based on methodological quality. Instead, the results of the critical appraisal were used to support the interpretation of findings, identify potential methodological limitations, and ensure transparency in evaluating the overall strength of the available evidence. Particular attention was given to methodological limitations that could affect the interpretation of specific findings, including the identification and control of confounding, the validity and reliability of exposure and outcome measurement, the completeness of follow-up, researcher reflexivity in qualitative studies, and the integration of qualitative and quantitative components in mixed-methods studies. These domain-level limitations were considered when interpreting the consistency and strength of evidence contributing to each synthesis category. Findings supported predominantly by studies with important methodological limitations were interpreted more cautiously, particularly where causal or directional relationships were suggested. To further inform the interpretation of the synthesis, the strength of evidence supporting each generic category was considered descriptively, taking into account the number and design of contributing studies, the methodological limitations identified through critical appraisal, the consistency of reported findings, and the directness of the evidence in relation to the category conclusions. This descriptive assessment was intended to contextualise the findings, but did not constitute a formal certainty-of-evidence assessment.

### 2.6. Data Synthesis

Given the substantial methodological and conceptual heterogeneity of the studies, statistical pooling was not considered appropriate. Inductive content analysis was selected because it enabled the integration of findings from quantitative observational, cohort, qualitative, and mixed-methods studies without requiring common effect measures or predefined synthesis categories. This approach was consistent with the exploratory aim of the review, which was not only to summarise reported findings but also to identify recurring patterns in the sources, consequences, and context-dependent effects of distraction-related phenomena across diverse forms of evidence.

To clarify the conceptual boundaries of the synthesis, the reported phenomena were distinguished by their operational role in each included study. External interruptions, environmental noise, alarms, and technological disturbances were considered potential sources of distraction, whereas multitasking and task switching were classified as work processes or responses to competing demands when reported in this manner. Mental workload, stress, situation awareness, emotional responses, and task performance were primarily considered outcome domains. Where a phenomenon fulfilled more than one role, its classification was based on the operational definition, study design, and reported relationships between variables in the original study. These distinctions were applied during the organisation and interpretation of the extracted findings rather than as predefined coding categories. 

Inductive content analysis was preferred over a formal convergent integrated mixed-methods synthesis because the primary objective was to identify and organise recurring conceptual patterns across heterogeneous findings rather than to transform different forms of evidence into a common data format. This approach enabled the comparison of findings based on conceptual similarity while preserving distinctions between their evidentiary characteristics. Following data extraction and methodological quality appraisal, the findings were synthesised using inductive content analysis, as described by Elo and Kyngäs [28]. The analysis followed the three main phases proposed by the authors: preparation, organisation, and reporting. During the preparation phase, the extracted findings from all studies were repeatedly read to achieve immersion in the data and identify meaningful units relevant to the review question. Quantitative findings, including observed frequencies, associations, task-performance outcomes, and other measured effects, were coded and considered alongside qualitative findings describing participants’ experiences and perceptions. For mixed-methods studies, quantitative and qualitative findings were considered according to the respective components of the study and their evidentiary characteristics.

During the organisation phase, open coding was performed by assigning descriptive codes to meaningful units. Similar codes were then compared and grouped into subcategories based on conceptual similarity. The subcategories were subsequently combined into broader generic categories, which were further abstracted into one overarching main category representing the central phenomenon across the studies. The analytical process was iterative, involving continuous comparison and refinement of codes and categories across studies while retaining information on the type and source of evidence contributing to each category. During the reporting phase, the resulting categories were described narratively and supported by evidence from the studies. The interpretation of each category took into account the study design, the nature of the reported evidence, and the methodological limitations identified during critical appraisal. Quantitative associations were distinguished from descriptive observational findings, while qualitative accounts were interpreted as evidence of participants’ experiences and perceptions rather than as confirmation of objectively measured effects. Theoretical explanations proposed by the original authors or developed during the review were distinguished from empirical findings. Consequently, the presence of findings from different study designs within the same conceptual category was not interpreted as indicating equivalent evidentiary strength or independent confirmation of the same effect. Conclusions were formulated cautiously, taking into account the nature of the evidence and the methodological limitations of the supporting studies. For the synthesis of context-dependent interruption effects, descriptions of interruptions as clinically relevant, functional, unnecessary, or disruptive were examined in relation to how they were reported in the original studies. These descriptions were interpreted based on their evidentiary basis, including clinicians’ reported perceptions, observed characteristics of interruptions, and the clinical functions attributed to interruptions. The resulting distinction between functional and potentially disruptive interruptions represents an interpretive synthesis rather than an objective classification based on standardised criteria. In particular, interruptions described as clinically relevant or initiated to prevent errors were not assumed to have demonstrated beneficial effects on patient outcomes.

Quantitative associations were not interpreted as evidence of causal relationships unless supported by the study design. Similarly, proposed cognitive mechanisms or implications for patient safety were treated as interpretive explanations when they had not been directly measured. The final synthesis provided a structured overview of the identified distraction factors, their consequences, and their relationships, while also highlighting gaps in the existing literature.

Cognitive ergonomics was not used as an a priori coding framework, as the synthesis remained inductive. Rather, after the categories were derived from the included studies, the cognitive ergonomics framework presented in Figure 1 was used as an interpretive lens to examine how the identified distraction-related phenomena were linked to cognitive demands, task and workflow characteristics, clinician responses, and broader work-system conditions. Sociotechnical systems thinking, organisational accident theory, and Safety-I/Safety-II were subsequently used as complementary perspectives for interpreting the organisational and adaptive dimensions of these relationships.

The initial coding and categorisation were performed by P.K. The developing coding structure, subcategories, and generic categories were subsequently reviewed by Z.F. and M.M. Differences in interpretation were discussed until consensus was reached.

## 3. Results

**Figure 2:**
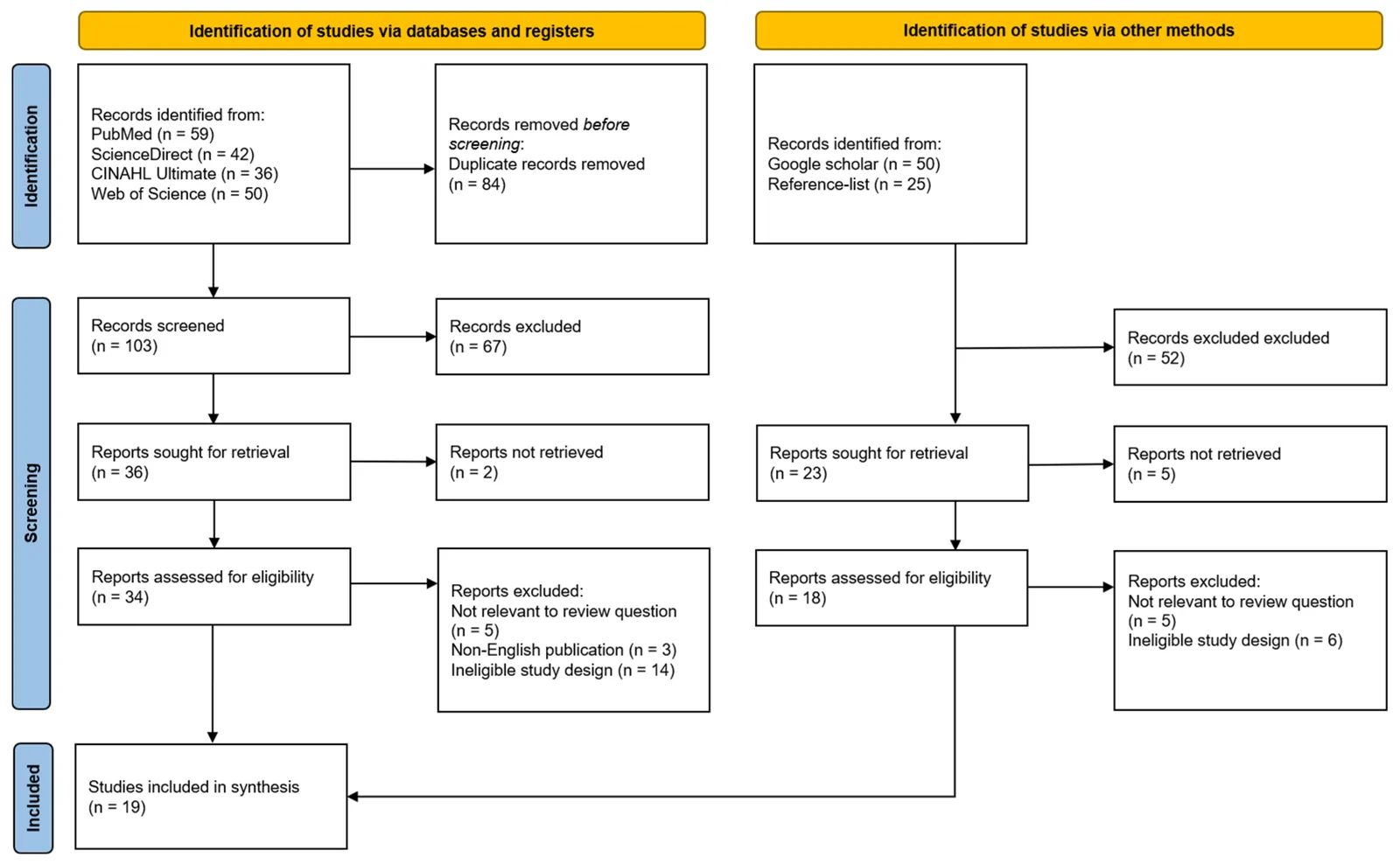
PRISMA flow diagram of the search process.

The study selection process was conducted in accordance with the PRISMA 2020 framework and is summarised in Figure 2.

A total of 187 records were identified through database searching, including 59 from PubMed, 50 from Web of Science, 42 from ScienceDirect, and 36 from CINAHL Ultimate. After removing 84 duplicate records, 103 records remained for title and abstract screening. Of these, 67 records were excluded because they did not meet the eligibility criteria. Thirty-six reports were sought for retrieval, of which two could not be retrieved. The remaining 34 full-text articles were assessed for eligibility. Following full-text assessment, 22 reports were excluded due to thematic irrelevance (*n* = 5), language-related eligibility criteria (*n* = 3), or inappropriate study design (*n* = 14). Consequently, 12 studies identified through database searching met the inclusion criteria.

An additional 75 records were identified through supplementary search methods, including Google Scholar (*n* = 50) and reference-list screening (*n* = 25). Following title and abstract screening, 52 records were excluded, and 23 reports were sought for retrieval. Five reports could not be retrieved, leaving 18 full-text reports for eligibility assessment. Following full-text review, 11 reports were excluded, because they were not relevant to the review question (*n* = 5) or had an ineligible study design (*n* = 6), resulting in seven additional studies being included in the review.

Overall, 19 studies met the predefined eligibility criteria and were included in the final synthesis. The study selection process and reasons for exclusion at the full-text stage are presented in the PRISMA 2020 flow diagram (Figure 2).

The studies varied in their methodological approaches, study populations, and the distraction-related factors investigated, reflecting the multidimensional nature of distraction in emergency department practice. To provide a structured overview of the evidence base and facilitate comparison across studies, the main methodological and substantive characteristics of the 19 studies are summarised in Table 2.

To further clarify the conceptual distinctions among distraction-related exposures, work processes, and outcomes, Table 3 presents the operational classification of the included studies by the constructs investigated and the outcome domains reported.

As shown in Table 3, the included studies primarily examined interruptions as potential sources of distraction, while multitasking and task switching were also investigated as work processes or responses to competing demands. The reported outcomes encompassed cognitive, emotional, and workflow-related domains, highlighting the conceptual heterogeneity of the evidence base.

### 3.1. Critical Appraisal of Studies

Most studies met the majority of the applicable appraisal criteria, although methodological limitations were identified in specific domains (Table 4, Table 5, Table 6 and Table 7). Among the quantitative observational studies assessed using the JBI Critical Appraisal Checklist for Analytical Cross-Sectional Studies, the inclusion criteria, the measurement of exposures and outcomes, the description of study settings and participants, and the statistical analyses were generally adequately reported.

Across the quantitative observational studies, the most frequently identified methodological limitations concerned the identification of potential confounding factors and the implementation of strategies to address them. These aspects were inconsistently reported, particularly in studies by Chisholm et al. [41,42], Kwon et al. [5], Kosits and Jones [46], Blocker et al. [34], Eng et al. [38], and Abu-Alhaija and Johnson [33], in which potential confounders were either not clearly identified or strategies to address them were insufficiently reported. The limited consideration of confounding in these studies reduces confidence that the reported associations represent independent effects of distraction-related phenomena and limits causal interpretation of the findings. These findings were therefore interpreted cautiously in the synthesis, particularly where associations between distraction-related phenomena and cognitive, workflow, or patient-safety outcomes were reported. Detailed appraisal results are presented in Table 4.

The cohort study by Lin et al. [37] identified methodological strengths in the validity and reliability of measurement procedures, as well as the appropriateness of statistical analysis (Table 5). However, potential confounding factors were not clearly identified or adequately addressed, representing an important methodological limitation.

Critical appraisal using the JBI Checklist for Qualitative Research indicated that Isbell et al. [31] met all applicable appraisal criteria, whereas the remaining three studies [36,43,44] demonstrated methodological limitations in specific appraisal domains (Table 6). Overall, the studies demonstrated good congruence between the research methodology, research questions, data collection procedures, data analysis, and interpretation of findings. Participants’ perspectives were adequately represented, ethical considerations were reported, and the presented data supported conclusions. The most consistently identified methodological limitations concerned the lack of an explicitly stated philosophical perspective and insufficient reporting of researcher reflexivity, including limited consideration of the researchers’ cultural or theoretical positioning and their potential influence on the research process.

Assessment using the Mixed Methods Appraisal Tool (MMAT, version 2018) indicated that all three mixed-methods studies [32,35,39] met both screening criteria and demonstrated methodological strengths across several appraisal domains (Table 7). The studies demonstrated clear research questions, an adequate rationale for using mixed-methods designs, integration of qualitative and quantitative components, and appropriate interpretation of the integrated findings. However, for all three studies, it was unclear whether divergences or inconsistencies between the quantitative and qualitative findings were adequately addressed (MM4). This represented the principal methodological limitation identified across the mixed-methods studies.

### 3.2. Main Findings of the Analysed Studies

The inductive content analysis resulted in one overarching main category: distraction-related phenomena and their consequences in emergency department work, comprising five generic categories and eleven interrelated subcategories: (1) sources and determinants of distraction factors; (2) workflow and task-performance consequences; (3) cognitive and emotional consequences and adaptation; (4) and (5) context-dependent effects of interruptions. The resulting analytical structure is presented in Table 8 [43,44,45,47,48].

Collectively, the findings indicate that distraction-related phenomena are multidimensional and embedded within the emergency department work system. Their occurrence and consequences were associated with communication processes, organisational and sociotechnical conditions, cognitive and emotional demands, workflow characteristics, and the clinical context in which interruptions occurred. Context-dependent effects of interruptions were also identified. However, the empirical evidence was unevenly distributed across these categories, with interruptions representing the dominant phenomenon across the studies, whereas other distraction-related dimensions were supported by substantially fewer studies.

#### 3.2.1. Sources and Determinants of Distraction Factors

The first generic category, sources and determinants of distraction factors, encompassed two subcategories: sources and types of interruptions and distractions and organisational and sociotechnical determinants of interruptions.

The first subcategory, sources and types of interruptions and distractions, showed that interruptions were consistently reported across emergency department settings and professional groups. They occurred predominantly during cognitively demanding and safety-critical activities, particularly triage, medication-related tasks, patient handovers, and sign-outs. These findings indicate that distraction-related phenomena were frequently observed during clinical activities requiring continuous attention, communication, and decision-making.

Communication emerged as the most consistently reported source of interruptions, being identified in seven studies, including interruptions initiated by patients and family members [5,30,32], healthcare professionals [5,30,32,39,46], and those related to communication and coordination processes [5,32,39,43,45,46]. Other sources included telephone- and beeper-related interruptions [5,32,35,38,43]; technology-related interruptions [35,38,43]; alarms [5]; technical disruptions [35]; environmental interruptions [34]; and self-interruptions [39]. Multitasking [40,41,42,44,45] and task switching [45] were also identified as work processes or responses through which healthcare professionals managed concurrent demands and interruptions. The second subcategory, organisational and sociotechnical determinants of interruptions, demonstrated that characteristics of the work system shaped exposure to interruptions. Relevant determinants included communication systems [32,43]; interprofessional communication [32,43,45]; role-dependent exposure to interruptions [39,45]; and physician seniority [45]. These findings suggest that interruptions should not be understood solely as isolated events but rather in relation to the organisational and sociotechnical conditions in which emergency care is delivered. The occurrence and sources of interruptions were documented across several observational studies, providing a comparatively broad empirical basis for describing interruption patterns. However, conclusions concerning organisational determinants were less robust, as several contributing studies [5,37,44,45,49] did not adequately identify or address potential confounding factors. Accordingly, these findings support the description of interruption sources and their organisational context more strongly than causal explanations of why interruptions occur.

#### 3.2.2. Workflow and Task-Performance Consequences

The second generic category, workflow and task-performance consequences, encompassed two subcategories: workflow disruption and task performance, and task delays and disruption of clinical activities. Together, these findings showed that interruptions affected both the continuity of workflow and the performance of specific clinical activities.

The first subcategory, workflow disruption and task performance, showed that interruptions were associated with workflow fragmentation [44,45,47] and with challenges related to resuming ongoing tasks. Task resumption following interruptions was identified [39], while difficulties in returning to interrupted tasks were also reported [44]. Interruptions and multitasking during handovers were additionally associated with information loss [44].

The second subcategory, Task delays and disruption of clinical activities, showed that interruptions were associated with delays and disruptions across several clinical activities. During triage, interruptions were associated with delays and disruptions of the triage process [30,33], while increased task duration was reported in other clinical activities [5,30]. Interruptions also occurred during documentation [45], prescribing [40], and direct patient care activities [5,30,33,39], indicating that their effects extended to both direct and indirect aspects of emergency department work. The strength of the evidence supporting this category varied depending on the outcomes examined. Directly observed task-related findings provided empirical support for associations between interruptions and workflow disruption, whereas qualitative accounts contributed contextual information about task fragmentation and resumption difficulties. However, the limited control of potential confounding factors in several contributing observational studies [5,36,44,45] restricts confidence in attributing these consequences specifically to interruptions. Therefore, the findings should be interpreted as associations and reported experiences rather than established causal effects.

#### 3.2.3. Cognitive and Emotional Consequences and Adaptation

The third generic category, cognitive and emotional consequences and adaptation, encompassed three subcategories: cognitive consequences for healthcare professionals, Interruption management and adaptation, and emotional responses and regulation. However, whereas cognitive consequences and interruption-management processes were represented across several studies, the emotional dimension was supported by only one qualitative study [31] and should therefore be considered exploratory.

The first subcategory, cognitive consequences for healthcare professionals, showed that interruptions and distraction-related demands were associated with increased subjective workload [32] and mental workload [32], as well as work-related stress [32]. Reduced situation awareness was also reported in association with higher interruption rates [35]. Findings from studies examining multitasking, interruptions, working-memory demands, and cognitive workload further suggested potential cognitive demands associated with interruption-rich work [40,44]. Interruptions and multitasking were also associated with increased task-error rates [40], indicating a potential relationship between interruption-related cognitive demands and task performance.

The second subcategory, interruption management and adaptation, showed that healthcare professionals used different strategies to manage interruptions and competing demands [45]. These included task switching and multitasking, as well as deferring, acknowledging, rejecting, or redirecting interruptions [45]. Responses to interruptions were context-dependent [45], while specific responses to interruptions during triage were also identified [30,33]. Task resumption following interruptions represented an additional aspect of interruption management [39]. A discrepancy between perceived and observed interruption frequency was also reported, indicating that healthcare professionals’ perceptions of interruption exposure did not always correspond with observed interruption frequency [38]. 

The third subcategory, emotional responses and regulation, was supported by one qualitative study [31]. Emotional triggers were identified at the patient, hospital, and broader healthcare system levels. Healthcare professionals reported both positive and negative emotional responses, as well as emotional strain that could influence clinical reasoning, communication, and patient engagement [31]. Emotion-regulation strategies included emotional suppression, cognitive reappraisal, and attentional distraction [31]. In the present synthesis, these emotional responses were not conceptualised as distraction factors themselves, but as psychological responses that may interact with cognitive demands and attentional processes. Because a single study supported this subcategory, these findings should be interpreted as an emerging dimension of cognitive and emotional demands in emergency department work. Overall, the evidentiary support within this category was uneven. Associations involving cognitive workload and situation awareness were examined in quantitative or mixed-methods studies [32,35,38], whereas interruption-management strategies were documented primarily through observational findings [48]. Although these studies provide empirical support for specific cognitive and behavioural responses, differences in measurement approaches and study designs limit their comparability. The qualitative evidence concerning emotional responses contributes an important experiential perspective but does not establish the frequency or magnitude of these effects across emergency department settings.

#### 3.2.4. Consequences for Patient Safety and Experience

The fourth generic category, consequences for patient safety and experience, encompassed two subcategories: patient safety consequences and patient experience and perceptions. The studies identified several potential patient-safety implications of interruptions and distraction-related phenomena, whereas evidence concerning patient experience was more limited and did not consistently demonstrate adverse effects. Of the 19 included studies, only one directly assessed task errors [43]. Other studies reported interruptions during medication-related activities [5,42,49], disruptions to triage processes [33,36], or information-flow difficulties during handovers [47], but these findings primarily served as indirect indicators of potential safety risks rather than as direct measures of medication errors or adverse patient outcomes.

The first subcategory, patient safety consequences, included several findings relevant to potential patient-safety risks. These included associations with increased task-error rates [40] and potential medication-safety concerns related to interruptions during medication-related activities [5,39]. Interruptions during triage were associated with delays and disruptions of the triage process [30,33], while interruptions and multitasking during handovers were associated with information loss [44]. Collectively, these findings indicate that interruption-related effects on workflow and task performance may have implications for patient safety. 

The second subcategory, patient experience and perceptions, was supported by limited evidence. One study directly examined patient-reported outcomes, including satisfaction with the triage experience and perceived professional competence, and found no significant association between nurses’ responses to interruptions and these outcomes [33]. A separate qualitative study reported healthcare professionals’ perceptions that emotional responses could influence communication and patient engagement [31]; however, these findings reflect clinicians’ perceptions rather than patient-reported or objectively measured patient outcomes. Accordingly, the available evidence does not support firm conclusions regarding the effects of distraction-related phenomena on patient experience. The overall evidentiary support for this category was limited by the scarcity of directly measured patient outcomes. Although task-error associations were reported [43], other contributing studies primarily examined workflow-related indicators, potential safety concerns, or clinicians’ perceptions [5,34,42,47]. Moreover, inadequate control of confounding in some observational studies [5] further limits causal interpretation. Consequently, evidence of task disruption or potential safety risks should not be equated with demonstrated patient harm.

#### 3.2.5. Context-Dependent Effects of Interruptions

The fifth generic category, context-dependent effects of interruptions, encompassed two subcategories: functional and clinically relevant interruptions and disruptive and potentially harmful interruptions. Together, these findings suggest that interruptions were not uniformly perceived or described as disruptive. Their reported functions and potential consequences varied with clinical relevance, timing, ongoing task demands, and healthcare professionals’ perspectives. These distinctions reflect the findings and interpretations reported in the original studies rather than objectively established categories of interruption effects.

The first subcategory, functional and clinically relevant interruptions, comprised interruptions that clinicians described as serving clinically relevant purposes or that were interpreted in the original studies as supporting emergency care processes [36,45]. These included: supporting timely information exchange [36,45]; facilitating care coordination and workload management [36,45]; being initiated to prevent errors [36]; and redirecting healthcare professionals toward higher-priority tasks [34,45]. Interruptions also supported information exchange across different clinical activities [36,39,45], with some interruption-related workflow processes associated with improved information exchange during discharge and transfer.

The second subcategory, disruptive and potentially harmful interruptions, encompassed interruptions perceived as unnecessary or disruptive by healthcare professionals, as well as interruptions occurring during activities in which workflow disruption or potential safety concerns were reported. Unnecessary interruptions were perceived as disruptive [39], whereas interruptions during high-risk activities, including medication-related tasks and triage, were associated with potential disruptions and safety concerns [5,30,33,39,40]. Interruptions and competing task demands were also associated with increased cognitive burden [40,44]. These findings indicate that the consequences of interruptions may depend on the context in which they occur, particularly on the clinical relevance and characteristics of the ongoing task. Evidence supporting the context-dependent functions of interruptions was primarily descriptive and observational, with qualitative findings providing insight into the perceived clinical relevance of information exchange and care coordination [39,48]. Although these findings suggest that some interruptions may serve useful clinical functions, they do not demonstrate that such interruptions independently improve patient safety or clinical outcomes. The evidence, therefore, supports a context-sensitive interpretation of interruptions, while conclusions concerning their potential benefits remain tentative.

## 4. Discussion

This systematic review suggests that distraction-related phenomena in emergency departments should not be understood as isolated events. Rather, their occurrence and consequences appear to emerge from interactions within a complex sociotechnical work system. 

Viewed through the conceptual framework presented in Figure 1, the findings suggest a dynamic pathway rather than a simple direct relationship between distraction and performance. Within the proposed cognitive-ergonomics framework, these processes are hypothesised to impose additional demands on attention, working memory, and situation awareness, potentially contributing to workflow disruption and task performance difficulties. However, these proposed cognitive pathways represent a theoretical interpretation of the observed findings rather than mechanisms directly established across the included studies.

These relationships appear to be moderated by contextual factors, including the timing and clinical relevance of the interruption, characteristics of the ongoing task, and clinicians’ adaptive responses. Accordingly, the framework conceptualises distraction-related phenomena as part of a dynamic work-system process in which the same interruption may have different consequences depending on its interaction with cognitive demands and the clinical context.

Across the studies, distraction-related phenomena were embedded within communication processes and broader organisational, technological, and environmental conditions. They were associated with cognitive and emotional responses, workflow consequences, and potential implications for patient safety and experience. From a cognitive ergonomics perspective, the impact of distraction-related phenomena therefore appears to depend not only on their frequency but also on their timing, clinical relevance, and interaction with ongoing task and cognitive demands. Sociotechnical systems thinking, organisational accident theory, and Safety-I/Safety-II perspectives are used as complementary explanatory frameworks that extend this interpretation beyond individual cognitive processes to encompass interactions among clinicians, tasks, technologies, organisational conditions, and adaptive work practices. These perspectives are therefore not treated as separate or competing theoretical frameworks, but as complementary levels of explanation within the broader cognitive-ergonomic interpretation of distraction-related phenomena in emergency care.

Although the review encompassed a broad range of distraction-related phenomena, interruptions were the most prominently represented phenomenon across the studies. This predominance may reflect, at least in part, greater research attention to observable interruption events than to less directly observable forms of distraction. Consequently, the current evidence base may provide a more developed understanding of interruptions than of the broader spectrum of distraction-related phenomena in emergency care. Accordingly, the broader distraction-related framework developed in this review should be interpreted as a conceptual synthesis of heterogeneous phenomena, while recognising that its empirical foundation is disproportionately concentrated on interruptions. Importantly, the descriptive assessment of evidence strength demonstrated that the five synthesis categories were not supported by evidence of equivalent methodological robustness. Direct observational evidence provided a comparatively stronger basis for describing interruption frequency and sources than for establishing their cognitive, performance-related, or patient-safety consequences. Findings concerning emotional responses, patient experience, and the potentially functional role of interruptions were more context-specific or supported by fewer studies. Consequently, the interpretation of these categories should reflect the nature and limitations of the underlying evidence rather than the frequency with which particular concepts appeared in the synthesis.

Whereas previous reviews have focused predominantly on specific aspects of interruptions, including their frequency and implications for medication safety [47], the present review extends this perspective by integrating multiple distraction-related phenomena within a cognitive ergonomics framework. Interruptions and competing task demands may place demands on limited attentional and working-memory resources, while multitasking and task switching represent work processes through which clinicians manage these concurrent demands [40,41,42,44,45]. From a cognitive-ergonomics perspective, these findings may be interpreted as suggesting that repeated shifts in attention may reduce the cognitive resources available for information integration, clinical reasoning, and decision-making [35,40,44]. However, these proposed mechanisms represent theoretical interpretations of the reported associations and qualitative findings, rather than cognitive pathways directly demonstrated by the included studies.

The complexity of emergency department work provides an important context for understanding these findings. High patient turnover, clinical uncertainty, time pressure, interprofessional coordination, and the simultaneous management of competing priorities create persistent cognitive demands that may increase clinicians’ vulnerability to interruption-related performance problems [32,34,36,38]. Across the studies, interruptions occurred during cognitively and clinically important activities, including medication-related tasks, documentation, triage, and patient handovers. Clinicians responded to interruptions through processes such as task switching and task resumption. In contrast, interruption exposure was associated across studies with increased cognitive workload, reduced situation awareness, and delayed task performance [30,35,39,40,44,45], while one study reported an association between interruptions and higher task-error rates [40]. These findings are consistent with the theoretical proposition that interruption-related consequences may vary according to the cognitive demands of the ongoing task. However, the underlying cognitive mechanisms were not consistently measured or directly tested across the included studies. This interpretation is compatible with Reason’s organisational accident theory, which conceptualises adverse outcomes as arising from interactions among multiple system vulnerabilities rather than from isolated individual failures [48]. From this perspective, interruptions may represent one contributing condition within a wider configuration of cognitive, organisational, technological, and environmental factors rather than an independent cause of error. This systems-based interpretation is also consistent with findings by Westbrook et al. [40], who found that interruptions, multitasking, fatigue, and lower working-memory capacity were associated with increased task-error rates among emergency physicians. Considered alongside findings related to handoffs, workload, and situational awareness [35,44], this suggests that interruption-related performance problems are unlikely to result from interruptions alone, but rather from their interaction with individual cognitive capacity, task demands, and organisational conditions.

Communication emerged as a prominent source of interruptions across the studies. Interruptions originated from patients and family members, healthcare professionals, and communication processes within the emergency department work [5,30,32,39,43,45,46]. Although communication-related interruptions may disrupt workflow and increase cognitive demands, they may also enable rapid information exchange, support care coordination, and facilitate timely redirection to higher-priority tasks when clinically relevant; some were also reported as being initiated with the aim of preventing errors [34,36,43,45]. Communication should therefore not be regarded solely as a source of distraction but as an essential component of emergency care whose disruptive or functional effect depends on its timing, relevance, and relationship to the ongoing task.

Importantly, interruptions should not be regarded as uniformly detrimental, nor should they be classified through a simple distinction between “beneficial” and “disruptive” interruptions. In the present synthesis, these distinctions were derived from descriptions and findings reported in the original studies, including clinicians’ subjective perceptions and the functions attributed to interruptions, rather than from a standardised or independently validated classification of interruption effects. Rather, interruptions should be understood as context-dependent events whose consequences emerge from the interaction between the interruption, the ongoing task, the recipient, and the broader work system. Factors such as clinical urgency, timing, task characteristics, information content, recipient workload, and organisational context may influence whether an interruption facilitates information exchange, care coordination, prioritisation, or error prevention, or instead contributes to cognitive and workflow disruption. Interruptions that convey urgent or clinically relevant information may support care coordination and timely redirection to higher-priority tasks and, in some contexts, be initiated to prevent errors [34,36,39,45]. Conversely, avoidable or poorly timed interruptions, particularly during cognitively demanding or safety-critical activities, may increase cognitive burden and disrupt ongoing clinical work. Accordingly, interruption frequency alone provides an incomplete indication of their potential consequences, and interruption-management strategies should not aim to eliminate interruptions indiscriminately but should reduce avoidable and low-value disruptions while preserving communication that supports safe and effective emergency care [49]. 

The findings can also be interpreted through established patient-safety and cognitive frameworks. Hollnagel’s Safety-I and Safety-II perspectives emphasise that safety in complex systems depends not only on preventing failure but also on understanding how professionals adapt to variability and maintain performance under dynamic conditions [50]. Similarly, the reported association between interruption frequency and reduced situation awareness [50] may be interpreted in relation to Endsley’s model [51], which emphasises the perception, comprehension, and anticipation of changes in the clinical environment. This theoretical perspective provides a possible explanation for the observed association but does not establish the cognitive mechanism underlying it [48,49,50,51].

The findings further support the principles of cognitive engineering, which emphasise that healthcare technologies, workflows, and information systems should be designed in line with human cognitive capabilities and limitations to support clinical decision-making and reduce the risk of errors in complex healthcare environments [51]. Because emergency care depends on continuous interprofessional collaboration, communication simultaneously supports safe patient care while creating opportunities for interruption-related cognitive burden.

The subjective experience of interruptions also appears to depend on more than their objective frequency. Eng et al. [38] identified substantial discrepancies between perceived and observed interruption frequency, suggesting that workload and task complexity may shape clinicians’ perceptions of distraction. Interruption characteristics also varied according to clinical location, task type, and workflow organisation, while structured group sign-outs were associated with fewer interruptions than individual sign-outs [34,37]. These findings highlight the importance of ergonomic work-system design that minimises avoidable cognitive burden while preserving effective interprofessional communication. 

An additional, although less extensively represented, dimension concerned emotional responses to the demands of emergency care. One qualitative study [31] identified emotional triggers arising from patient encounters, organisational conditions, and broader system-level factors, as well as positive and negative emotional responses and emotion-regulation strategies. These responses were reported as potentially influencing clinical reasoning, communication, and patient engagement. In the present review, emotional responses were not conceptualised as distraction factors themselves, but as psychological responses that may interact with cognitive demands and attentional processes. Given that a single study supported this dimension, these findings should be considered exploratory and warrant further investigation.

Evidence concerning patient-safety consequences was considerably more limited than evidence concerning interruption frequency, cognitive workload, situation awareness, and workflow disruption. Among the 19 included studies, only Westbrook et al. [40] directly examined task errors, reporting associations with interruptions, multitasking, fatigue, and working-memory capacity. Other studies documented interruptions during medication-related activities [5,39,40,44], triage disruption [30,33], and information-flow difficulties during handovers [44]. Although these findings identify circumstances that may be relevant to patient safety, they do not constitute direct evidence of medication errors, adverse events, or patient harm. Accordingly, the available evidence supports concern about potential safety implications but does not establish that distraction-related phenomena independently cause adverse patient outcomes.

Evidence concerning patient experience was comparatively limited. One study directly examined patient-reported outcomes, including satisfaction with the triage experience and perceived professional competence, and found no significant association between nurses’ responses to interruptions and these outcomes [33]. In contrast, a qualitative study reported healthcare professionals’ perceptions that emotional responses could influence communication and patient engagement [31]. These findings represent clinician-reported perceptions rather than direct patient-reported or objectively measured patient outcomes. Therefore, the available evidence is insufficient to draw firm conclusions regarding the effects of distraction-related phenomena on patient experience.

The predominance of observational designs and methodological heterogeneity limits the strength of conclusions that can be drawn regarding causal relationships between distraction factors and clinical outcomes. Studies differed in professional groups, clinical activities, definitions and measurement of interruptions and distractions, and outcomes examined, which also limits direct comparison across studies. The broad temporal range of the studies should also be considered when interpreting these findings, as changes in digital technologies, documentation systems, communication tools, and workflow organisation over the past two decades may have altered the nature and management of distraction-related phenomena in emergency care. Furthermore, some dimensions identified in the synthesis, particularly emotional consequences and patient experience, were supported by relatively few studies. Consequently, findings derived from studies with important methodological limitations or from a limited number of qualitative investigations should be interpreted with greater caution than findings supported by a broader and more directly measured empirical evidence base. The apparent prominence of interruptions may therefore reflect both their importance in emergency care and the greater methodological attention they have received compared with less observable forms of distraction. Overall, the findings support a systems-oriented approach to distraction-related phenomena in emergency care. Potential cognitive-ergonomic strategies may include improvements in work-system design, communication practices, and technologies intended to reduce unnecessary cognitive burden while supporting effective coordination and information exchange. However, their effectiveness requires empirical evaluation. Future research should use clearer distinctions among interruptions, distractions, multitasking, and task switching and further examine under-researched cognitive, emotional and patient-related outcomes.

### 4.1. Limitations

Several limitations should be acknowledged. Most studies used observational designs, which limit causal inference and may be susceptible to observer effects, whereby the presence of an observer influences healthcare professionals’ behaviour [29,34,35]. Confidence in the strength of the synthesis is further limited by recurring methodological weaknesses in several observational studies, particularly the inadequate identification and control of potential confounding factors. Consequently, reported associations between distraction-related phenomena and cognitive, workflow, or patient-safety outcomes should be interpreted primarily as observational associations rather than evidence of independent or causal effects. Furthermore, the cognitive mechanisms proposed within the interpretive framework, particularly those involving attentional resources, working memory, information integration, and clinical decision-making, were not consistently measured or directly tested in the included studies and should therefore be regarded as theoretically informed explanations rather than empirically established causal pathways. The identification and classification of interruptions may also involve subjective judgement, particularly when operational definitions differ across studies [33]. In addition, relatively short observation periods may not adequately capture variations across shifts, workload conditions, weekdays, or seasonal patterns. Some studies included relatively small professional samples, such as the triage observation study by Johnson et al. [30], further limiting the transferability of findings across emergency department settings. Together, these methodological characteristics constrain the extent to which findings can be generalised across institutions and healthcare systems [35]. 

Methodological limitations of the present review should also be considered. The review was not prospectively registered, and no standalone formal review protocol was prepared. Although backward citation searching was performed, through manually screening the reference lists of eligible articles and relevant review papers, forward citation searching was not conducted. Furthermore, the inclusion of a mandatory outcome-related search block focusing on stress and patient care may have limited the search strategy’s sensitivity and led to the omission of relevant studies addressing distraction-related phenomena that did not explicitly refer to these outcomes. Additionally, the inclusion of “cognitive load” among the distraction-related search terms is a conceptual limitation, as cognitive workload was considered primarily a potential consequence rather than a distraction factor in this review.

Seven reports in total could not be retrieved for full-text assessment, including two reports identified through database searching and five through supplementary searches. Consequently, their eligibility could not be determined, and the possibility that relevant evidence was excluded introduces a potential source of selection bias that may have affected the synthesis’s comprehensiveness and representativeness. Although screening and eligibility assessment were conducted independently by three reviewers and disagreements were resolved through consensus, inter-rater agreement was not formally quantified, for example, using an appropriate inter-rater agreement statistic. This limits the ability to quantify the consistency of reviewers’ screening decisions, although independent screening and consensus procedures were used to reduce selection bias.

Considerable conceptual, methodological, and contextual heterogeneity represents an important limitation of the evidence base. Study designs, definitions and measurement approaches for interruptions and other distraction-related phenomena, clinical activities, professional groups, and reported outcomes varied substantially across studies. This heterogeneity limited direct comparison of findings and precluded quantitative synthesis. Consequently, pooled effect sizes could not be estimated, limiting the ability to quantify the magnitude and consistency of associations across studies and reducing the certainty of the overall evidence. Although findings from different study designs were integrated into common conceptual categories, their evidentiary strength varied, and the inductive synthesis did not include a formal assessment of the overall certainty of evidence for each category. Consequently, the resulting categories should be interpreted as a conceptual organisation of heterogeneous findings rather than as confirmation of consistently established effects.

Contextual heterogeneity should also be considered, as the studies were conducted across different countries and healthcare systems and involved different professional groups, emergency department configurations, organisational practices, and work cultures. 

Moreover, inconsistent and partly overlapping terminology concerning interruptions, distractions, multitasking, and task switching may have influenced how distraction-related phenomena were identified, classified, and synthesised in the present review. Furthermore, the distinction between functional and disruptive interruptions was partly based on clinicians’ subjective perceptions and interpretations reported in the original studies, rather than on consistently applied objective criteria. Publication bias cannot be excluded, and restricting the review to English-language publications may have introduced language bias. Given the international diversity of emergency care organisations, communication practices, technologies, and work-system characteristics, this restriction may limit the findings’ international representativeness and transferability. Furthermore, grey literature, dissertations and theses, conference proceedings, preprints, and unpublished studies were not systematically searched, which may have increased the risk of publication bias and resulted in potentially relevant evidence being missed.

The evidence base was also unevenly distributed across the identified dimensions. Interruptions were substantially more frequently represented than other distraction-related phenomena, whereas evidence concerning emotional demands and patient experience was comparatively limited. Consequently, the prominence of interruptions in the synthesis may reflect not only their relevance to emergency care but also the greater research attention they have received. Findings related to less extensively studied dimensions should therefore be interpreted with caution. 

An additional limitation concerns the temporal heterogeneity of the evidence, with studies spanning from 2000 to 2024. During this period, emergency department work systems have evolved substantially, particularly regarding digital documentation, electronic health records, communication technologies, monitoring systems, and technology-mediated workflows. These changes may have altered both the sources and patterns of interruptions and distractions, as well as the strategies healthcare professionals use to manage them. Consequently, findings from earlier studies may not be fully transferable to contemporary, increasingly digital emergency care environments. The synthesis should therefore be interpreted in light of this temporal heterogeneity.

### 4.2. Implications for Practice, Organisation, and Future Research

The findings indicate that not all interruptions in emergency departments can or should be eliminated. Interruption-management strategies should instead focus on preventing or minimising avoidable and low-value disruptions while preserving clinically necessary interruptions that support timely information exchange and care coordination. Based on the patterns identified in this review, potential strategies for future evaluation may include protected periods for high-risk activities, structured handovers, interruption-aware triage processes, and workflow redesign to reduce unnecessary cognitive burden [30,37,40,43,50]. However, the effectiveness of these approaches in reducing interruption-related risks was not directly established by the evidence synthesised in this review and requires evaluation in prospective intervention studies.

Education and professional training may complement organisational interventions by strengthening clinicians’ competencies in interruption management, task prioritisation, situation awareness, and recognition of cognitive workload. Training should not focus solely on avoiding interruptions but also on developing the ability to assess their urgency and clinical relevance, decide whether to accept, defer, redirect, or reject an interruption, and resume interrupted tasks safely. Simulation-based learning is a potential approach to practising these competencies under realistic conditions involving competing demands, time pressure, multitasking, and clinically relevant interruptions, although its effectiveness in improving interruption management and patient safety outcomes requires empirical evaluation. Such approaches may also support team communication, shared awareness, and adaptive coordination in dynamic emergency department environments. However, the effectiveness of specific educational and simulation-based interventions in reducing interruption-related cognitive and safety risks was not established by the studies included in this review and should therefore be evaluated in future research. At the organisational level, cognitive-ergonomic redesign of communication processes, digital technologies, and workflows may help reduce unnecessary interruptions, support efficient information exchange, and potentially contribute to safer care. Rather than focusing exclusively on reducing interruption frequency, future intervention studies could examine context-sensitive approaches that account for interruption urgency, clinical relevance, timing, task complexity, and concurrent cognitive demands [17,40,43,49,50]. Future intervention studies should evaluate whether combining workflow redesign, structured communication practices, and technologies that prioritise clinically important information can reduce unnecessary cognitive burden while preserving adaptive team performance.

From a practical perspective, the findings identify several areas that may warrant evaluation in future intervention studies, particularly during safety-critical activities such as medication preparation, triage, and patient handovers. Potential approaches include: interruption-aware communication protocols; optimisation of alarm management systems; workflow redesign; and staff education. These strategies should be regarded as theoretically and empirically informed directions for future evaluation rather than interventions whose effectiveness has been established by the present review. Future intervention studies should assess whether such approaches can reduce unnecessary cognitive and workflow disruption while preserving clinically relevant communication and coordinated patient care.

Future research should use clearer and more standardised definitions of interruptions, distractions, multitasking, and task switching to improve comparability across studies. Greater attention should also be given to less-studied dimensions, including emotionally mediated and internally mediated distractions, as well as patient-reported outcomes. Intervention studies are needed to evaluate cognitive-ergonomic strategies in real-world emergency settings using standardised outcomes that capture cognitive workload, interruption characteristics, workflow performance, clinician performance, patient experience, and patient safety.

## 5. Conclusions

This systematic review indicates that distraction-related phenomena are common features of emergency department work and arise from interactions among communication processes, organisational demands, technologies, environmental conditions, and competing clinical priorities. Interruptions were the most prominently represented phenomenon and were associated with workflow disruption, increased cognitive workload, heightened attentional demands, and reduced situation awareness. However, direct evidence concerning task errors was limited to one included study, while evidence concerning medication-related errors and adverse patient outcomes was largely indirect or absent. Consequently, the findings do not establish a causal relationship between distraction-related phenomena and patient harm.

The findings support context-sensitive interruption management rather than strategies focused solely on reducing interruption frequency. From a cognitive ergonomics perspective, workflow design, communication practices, and technology should aim to reduce unnecessary cognitive burden while preserving clinically relevant communication and coordination. However, the heterogeneity and predominantly observational nature of the available evidence limit causal inference. Future research should therefore adopt clearer and more standardised definitions of distraction-related phenomena and evaluate context-sensitive cognitive-ergonomic interventions in real-world emergency settings.

## Figures and Tables

**Table 1:** Inclusion and exclusion criteria.

Category	Inclusion Criteria	Exclusion Criteria
Population	Healthcare professionals employed in the emergency department	Healthcare professionals not working in emergency departments
Interest	Distraction-related phenomena affecting healthcare professionals, including interruptions, environmental or technology-related distractions, competing clinical demands, workflow disruptions, and related work processes such as multitasking and task switching	Studies examining mental workload, stress, emotional responses, situation awareness, or other cognitive or performance outcomes without an identifiable distraction-related phenomenon, interruption, competing demand, or related work process
Outcome	Outcomes related to cognitive functioning, workflow or task performance, emotional responses, healthcare professional well-being, patient experience, or patient safety.	Studies not reporting outcomes relevant to cognitive functioning, workflow or task performance, emotional responses, healthcare professional well-being, patient experience, or patient safety.
Study type	Full-text primary quantitative, qualitative, and mixed-methods research articles published in peer-reviewed scientific journals	Systematic and other reviews, grey literature, dissertations and theses, conference abstracts and proceedings, preprints, opinion papers, commentaries, and unpublished studies
Language	English	Studies not published in English

**Table 2:** Characteristics of Studies.

Author (Year); Country	Study Design	Aim	Sample (*n*)	Main Findings
Forsyth et al. [29]; USA	Quantitative, retrospective observational study	To describe interruptions experienced by nurses and examine their association with workload.	Nurses (*n* = 38)	A total of 3229 interruptions were recorded (85 per shift; 8.7/h). Interruptions were moderately associated with increased objective workload indicators and nurses’ subjective mental strain, suggesting a measurable cognitive burden associated with workflow disruptions.
Johnson et al. [30]; USA	Quantitative, prospective observational study	To identify sources and causes of triage interruptions and their impact.	Nurses (*n* = 8)	Interruptions occurred frequently during Emergency Department (ED) triage and were associated with disruptions to the triage process. Interruptions originated from multiple sources, including staff communication, patient- or family-related demands, and operational issues. Although interruption frequency differed between nurses, triage duration did not differ significantly. These findings indicate that triage interruptions represent a workflow and cognitive-ergonomic challenge, not only a time-efficiency issue.
Isbell et al. [31]; USA	Qualitative, semi-structured interviews	To explore emotional experiences and their impact on decision-making.	Nurses (*n* = 41); physicians (*n* = 45)	ED physicians and nurses reported a wide range of positive and negative emotions triggered by patient-related, hospital-level, and system-level factors. Negative emotions were perceived as potentially influencing clinical decision-making, communication, patient engagement, and patient safety. Providers reported emotion-regulation strategies, including suppression, distraction, and cognitive reappraisal.
Weigl et al. [32], Germany	Mixed-methods observational study	To identify the sources and contents of workflow interruptions and examine their associations with work stress.	ED physicians and nurses; 80 observation sessions (50 nurses, 30 physicians); 77 matched observation and self-evaluation sessions; 116 h 51 min of observation	A total of 877 workflow interruptions were observed, with a mean rate of 7.51 interruptions/h. Interruptions were most frequently caused by colleagues from another profession (27.1%), colleagues from the same profession (24.1%), and telephone/beeper events (21.0%). Interruptions related to parallel cases were significantly associated with increased work stress (β = 0.24; *p* = 0.03), whereas most other interruption types were not associated with stress.
Abu-Alhaija & Johnson [33]; USA	Quantitative, prospective observational study	To examine the relationship between interruption response and patient perception.	Nurses (*n* = 51)	Of 93 observed triage interviews conducted by 51 nurses, 66 were interrupted. Triage interruptions were common and most often resulted in delays in care. No significant association was found between nurses’ responses to interruptions and patients’ perceived confidence in nurses’ technical skills, perceived competence of the triage nurse, or satisfaction with the triage experience. Nurses’ demographic characteristics were not associated with their responses to interruptions.
Blocker et al. [34]; USA	Quantitative, prospective observational study	To evaluate the characteristics of workflow interruptions experienced by academic emergency physicians.	28 attending emergency physicians; 28 shifts; 210 clinical hours of observation	A total of 2355 interruptions were recorded (11.21 interruptions/h). Face-to-face interruptions by physicians were the most frequent (26.0%), followed by nurse interruptions (21.7%) and environmental interruptions (20.8%). Interruption duration increased significantly with interruption priority (*p* < 0.001). Patient rooms were identified as high-risk locations for low-priority interruptions.
Weigl et al. [35], Germany	Mixed-methods study	To examine how the sources and contents of workflow interruptions influence situation awareness among ED physicians and nurses.	ED physicians and nurses #; 74 observation sessions; 110 h 40 min of observation	A total of 1205 workflow interruptions were observed (10.9 interruptions/h). Higher interruption rates were associated with lower situation awareness (β = −0.27). Interruptions related to telephone/beeper events, technical malfunctions, and communication concerning completed cases were particularly associated with lower situation awareness.
Berg et al. [36]; Sweden	Qualitative study (interviews)	To explore clinicians’ reasons for interrupting colleagues during ED work	Clinicians (*n* = 20) (10 physicians and 10 registered nurses) from 2 EDs	Three main reasons for interrupting colleagues were identified: working conditions, improving the initiator’s work process, and influencing colleagues’ work processes. Several interruptions were initiated to prevent mistakes, provide relevant information, and improve patient care. Interruptions perceived negatively by recipients were mainly related to working conditions.
Lin et al. [37]; USA	Quantitative, prospective observational cohort study	To determine the frequency and classification of interruptions during Emergency Medicine (EM) resident sign-out before and after implementation of a group sign-out process.	123 sign-out observations: 58 individual and 65 group sign-outs	Group sign-outs had significantly fewer interruptions per minute than individual sign-outs (0.05 vs. 0.16; *p* < 0.001), as well as fewer total interruptions (44 vs. 73; *p* = 0.007), non-emergent interruptions (38 vs. 67; *p* = 0.005), and in-person interruptions (14 vs. 44; *p* < 0.001). Group sign-outs were longer but were associated with fewer interruptions.
Eng et al. [38]; USA	Quantitative observational study	To compare EM physicians’ perceptions of distractions and interruptions with their actual occurrence in the ED.	Physicians (*n* = 38); residents (*n* = 23)	A total of 2438 overhead pages were recorded (23.2 per shift), compared with a perceived rate of 43.2 per shift. In addition, 333 individual interruptions were observed (4.26 per shift), whereas providers perceived 53.5 interruptions per shift. Providers, therefore, substantially overestimated both distractions and individual interruptions. Attending physicians perceived and experienced more interruptions than residents.
Berg et al. [39]; Sweden	Mixed-methods study	To explore the frequency, types, and perceptions of interruptions during emergency department work.	Clinicians (*n* = 18)	Clinicians experienced approximately 5.1 interruptions/h, most commonly during information exchange activities. Medication preparation was among the most frequently interrupted tasks. Most interruptions were resumed, and interruptions were perceived negatively, primarily when they disrupted workflow or were considered unnecessary.
Westbrook et al. [40]; Australia	Prospective observational study	To examine the association between interruptions, multitasking, fatigue, working memory capacity, and task errors among emergency physicians.	Emergency physicians (*n* = 36)	Task errors were significantly associated with interruptions, multitasking, fatigue, and lower working memory capacity, suggesting important cognitive mechanisms that underlie patient safety risks in emergency care.
Chisholm et al. [41]; USA	Observational time-motion study	To determine the number and types of interruptions experienced by emergency physicians during ED work.	Emergency physicians (*n* = 10)	Emergency physicians experienced frequent interruptions and multitasking demands, with a mean of 30.9 interruptions per 180-min observation period, supporting the view of ED work as highly interruption-driven.
Chisholm et al. [42]; USA	Observational study	To compare workplace interruptions and multitasking between emergency physicians and primary care physicians.	Emergency physicians (*n* = 9) and Primary care physicians (*n* = 9).	Emergency physicians experienced more interruptions and managed more patients concurrently than primary care physicians, indicating important ergonomic differences between emergency medicine and primary care work environments.
Brixey et al. [43]; USA	Observational case study	To characterise the frequency, sources, and patterns of interruptions in a level one trauma centre.	Emergency physicians (*n* = 5) and registered nurses (*n* = 8)	Interruptions occurred frequently and were primarily associated with communication and coordination activities. Most interruptions originated from physicians, nurses, and communication technologies, demonstrating the interruption-rich and cognitively demanding nature of trauma and emergency care environments.
Laxmisan et al. [44]; USA	Qualitative ethnographic observational and interview study	To examine multitasking, interruptions, decision-making, and cognitive demand during and after team handoffs in emergency care.	Six ED clinicians (physicians, residents, and nurses) were interviewed; the number of clinicians observed was not reported.	Interruptions were prevalent and diverse. Attending physicians were interrupted approximately every 9 min, and residents every 14 min. Multitasking and shift changes contributed to gaps in information flow and increased cognitive demand, indicating potential patient-safety risks in emergency care.
Kwon et al. [5], South Korea	Quantitative descriptive observational study	To examine the degree of interruptions experienced by emergency department nurses and related factors.	Nurses (*n* = 23); 120 h of observation	Interruptions occurred 6.4 times/h and represented 9.1% of nursing task time. Communication with patients, families, and nurses was the primary source of interruptions. The longest duration and highest frequency of interruptions occurred during medication tasks. Interruptions significantly increased the time required for selected nursing tasks.
Walter et al. [45], Australia	Observational workflow time study	To examine how emergency doctors manage competing workload demands and interruptions in an interruptive emergency department environment.	36 emergency doctors; 121.7 h of observation; 965 prompts/interruptions recorded.	Task-switching was the most frequently used strategy for responding to interruptive prompts. Prompt rates and response strategies varied according to task type, prompt type, location, and physician seniority. Most prompts involved communication from nurses or doctors and were particularly frequent during documentation and other non-verbal tasks. Communicative prompts played an important role in patient care and workload management, suggesting that the benefits of timely information transfer and advice may offset the risks associated with interruptions.
Kosits et al. [46]; USA	Descriptive observational study	To determine the frequency and types of interruptions among ED nurses and the percentage occurring during medication-related activities.	Nurses (*n* = 30) from three EDs; 60 h of observation	A total of 200 interruptions were observed (3.3 interruptions/h per RN). Most interruptions (95%) involved face-to-face communication, while 27.5% occurred during medication-related activities. The findings demonstrate frequent interruptions in ED nursing practice, including during medication-related activities.

Note: *n*—number; ED—Emergency Department; EM—Emergency Medicine; #—number of participants not reported; RN—registered nurses.

**Table 3:** Classification of included studies by distraction-related constructs and outcome domains.

Study	Distraction-Related Construct	Outcome Domain/Endpoint
Forsyth et al. [32]	Interruptions (E)	Workload; mental strain
Johnson et al. [33]	Triage interruptions (E)	Workflow; triage time
Isbell et al. [34]	Emotional triggers (C); distraction as coping (R)	Emotions; perceived care effects
Weigl et al. [35]	Workflow interruptions (E)	Work stress
Abu-Alhaija & Johnson [36]	Triage interruptions (E); responses (R)	Delays; patient perceptions
Blocker et al. [37]	Workflow interruptions (E)	Interruption characteristics
Weigl et al. [38]	Workflow interruptions (E)	Situation awareness
Berg et al. [39]	Interpersonal interruptions (E)	Workflow; perceived care effects
Lin et al. [40]	Sign-out interruptions (E)	Frequency; sign-out duration
Eng et al. [41]	Pages; interruptions (E)	Observed/perceived frequency
Berg et al. [42]	Task interruptions (E)	Task resumption; workflow
Westbrook et al. [43]	Interruptions (E); multitasking (P)	Task errors
Chisholm et al. [44]	Interruptions (E); multitasking (P)	Frequency: work patterns
Chisholm et al. [45]	Interruptions (E); multitasking (P)	Frequency; concurrent workload
Brixey et al. [46]	Communication/technology interruptions (E)	Frequency; sources
Laxmisan et al. [47]	Handoff interruptions (E); multitasking (P)	Information flow; cognitive demand
Kwon et al. [5]	Communication interruptions (E)	Task duration; frequency
Walter et al. [48]	Interruptive prompts (E); task switching (R)	Response patterns; workload
Kosits et al. [49]	Face-to-face interruptions (E)	Frequency; task distribution

Note. E = exposure/potential distraction source; P = work process; R = response; C = contextual trigger. Constructs were classified according to their operational role in the original studies. Outcome domains include both measured or reported outcomes and descriptive study endpoints; their classification does not imply causal relationships.

**Table 4:** Critical Appraisal of Quantitative Observational Studies Using the JBI Checklist for Analytical Cross-Sectional Studies.

Author (Year)	Q1	Q2	Q3	Q4	Q5	Q6	Q7	Q8
Forsyth et al. [29]	Y	Y	Y	Y	U	N	Y	Y
Johnson et al. [30]	Y	Y	Y	Y	Y	Y	Y	Y
Abu-Alhaija & Johnson [33]	Y	Y	Y	Y	N	N	Y	Y
Blocker et al. [34]	Y	Y	Y	Y	N	N	Y	Y
Eng et al. [38]	Y	Y	Y	Y	N	N	Y	Y
Westbrook et al. [40]	Y	Y	Y	Y	Y	Y	Y	Y
Chisholm et al. [41]	U	Y	Y	Y	N	N	Y	Y
Kwon et al. [5]	Y	Y	Y	Y	N	N	Y	Y
Walter et al. [45]	Y	Y	Y	Y	Y	Y	Y	Y
Kosits & Jones [46]	Y	Y	Y	Y	N	N	Y	Y
Chisholm et al. [42]	Y	Y	Y	Y	N	N	Y	Y

Note: Y—Yes; N—No; U—Unclear; Q1—inclusion criteria clearly defined; Q2—valid measurement of condition; Q3—valid measurement of exposure; Q4—valid measurement of outcomes; Q5—confounders identified; Q6—Strategies for confounders; Q7—appropriate statistical analysis; Q8—detailed description of subjects/setting.

**Table 5:** Critical Appraisal of the Cohort Study Using the JBI Checklist.

Author (Year)	Q1	Q2	Q3	Q4	Q5	Q6	Q7	Q8	Q9	Q10	Q11
Lin et al. [37]	Y	Y	Y	N	N	Y	Y	U	U	NA	Y

Note: Y—Yes; N—No; U—Unclear; NA—not applicable; Q1—similar groups/recruited from same population; Q2—exposure measured similarly; Q3—exposure measured validly and reliably; Q4—confounding factors identified; Q5—strategies to deal with confounding; Q6—participants free of outcome at baseline; Q7—outcomes measured validly and reliably; Q8—follow-up time sufficient; Q9—follow-up complete/adequately described; Q10—strategies to address incomplete follow-up; Q11—appropriate statistical analysis.

**Table 6:** Critical Appraisal of Qualitative Studies Using the JBI Checklist for Qualitative Research.

Author (Year)	Q1	Q2	Q3	Q4	Q5	Q6	Q7	Q8	Q9	Q10
Isbell et al. [31]	Y	Y	Y	Y	Y	Y	Y	Y	Y	Y
Berg et al. [36]	U	Y	Y	Y	Y	N	N	Y	Y	Y
Brixey et al. [43]	U	Y	Y	Y	Y	N	N	Y	Y	Y
Laxmisan et al. [44]	U	Y	Y	Y	Y	N	N	Y	Y	Y

Note: Y—Yes; N—No; U—Unclear; Q1—congruity between philosophical perspective and methodology; Q2—congruity between methodology and research question; Q3—congruity between methodology and data collection methods; Q4—congruity between methodology and representation/analysis of data; Q5—congruity between methodology and interpretation of results; Q6—researcher located culturally/theoretically; Q7—influence of researcher on research addressed; Q8—participants and their voices adequately represented; Q9—ethical approval evident; Q10—conclusions flow from analysis/data.

**Table 7:** Critical Appraisal of Mixed-Methods Studies Using the MMAT.

Author (Year)	S1	S2	MM1	MM2	MM3	MM4	MM5
Weigl et al. [32]	Y	Y	Y	Y	Y	U	Y
Weigl et al. [35]	Y	Y	Y	Y	Y	U	Y
Berg et al. [39]	Y	Y	Y	Y	Y	U	Y

Note: Y—Yes; U—Unclear; S1—clear research questions; S2—data address research questions; MM1—adequate rationale for mixed-methods design; MM2—effective integration of qualitative and quantitative components; MM3—adequate interpretation of integrated outputs; MM4—divergences/inconsistencies adequately addressed; MM5—quality criteria of each tradition met.

**Table 8:** Inductive Content Analysis.

Main Category	Generic Category	Subcategory	Codes
Distraction-related phenomena and their consequences in emergency department work	Sources and determinants of distraction factors	Sources and types of interruptions and distractions	Physician interruptions [34,40,41,42,45]; nurse interruptions [5,30,32,33,39,46]; triage interruptions [30,33]; handover/sign-out interruptions [37]; patient/family interruptions [5,30,32]; staff-initiated interruptions [5,30,32,39,43,46]; communication-related interruptions [5,32,39,43,45,46]; coordination-related interruptions [43,45]; telephone/beeper interruptions [5,32,35,38,43]; technology-related interruptions [35,38,43]; alarms [5]; technical disruptions [35]; environmental interruptions [34]; self-interruptions [39]; medication-related interruptions [5,39,46]; multitasking [40,41,42,44,45]; task switching [45].
		Organisational and sociotechnical determinants of interruptions	Communication systems [32,43]; role-dependent exposure [39,45]; physician seniority [45]; interprofessional communication [32,43,45].
	Workflow and task-performance consequences	Workflow disruption and task performance	Workflow fragmentation [41,42,44]; resumption difficulties [44].
		Task delays and disruption of clinical activities	Triage delays/disruption [30,33]; increased task duration [5,30]; documentation disruption [45]; prescribing disruption [40]; direct-care disruption [5,30,33,39].
	Cognitive and emotional consequences and adaptation	Cognitive consequences for healthcare professionals	Increased work stress [32]; reduced situation awareness [35]; increased cognitive demands [44]; working-memory demands [40,44]; cognitive burden [40,44]; increased subjective workload [29]; increased mental workload [29].
		Interruption management and adaptation	Interruption-management strategies [45]; responses to interruptions [30,33]; task resumption [39]; task switching [45]; multitasking [45]; interruption deferral [45]; interruption acknowledgement [45]; interruption rejection/deflection [45]; perceived–observed discrepancy [38]; context-dependent responses [45].
		Emotional responses and regulation	Patient-related emotional triggers [31]; hospital-level emotional triggers [31]; system-level emotional triggers [31]; positive and negative emotions [31]; emotional strain [31]; effects on clinical reasoning [31]; effects on communication and patient engagement [31]; emotional suppression [31]; cognitive reappraisal [31]; attentional distraction [31].
	Consequences for patient safety and experience	Patient safety consequences	Task errors [40]; potential medication-safety concerns [5,39]; information loss during handovers [44].
		Patient experience and perceptions	Triage satisfaction [33]; perceived professional competence [33]; communication and patient engagement [31].
	Context-dependent effects of interruptions	Functional and clinically relevant interruptions	Clinically relevant interruptions [36,45]; interruptions initiated to prevent errors [36]; timely information exchange [36,45]; priority redirection [34,45]; care coordination [36,45]; workload management [36,45].
		Disruptive and potentially harmful interruptions	Unnecessary interruptions [39]; interruptions during high-risk tasks [5,30,33,39,40].

## Data Availability

No new primary data were generated in this systematic review. All data analysed in this study were obtained from previously published studies and are reported within the article and its Supplementary Materials.

## Supplementary Materials

The following supporting information can be downloaded at: https://www.mdpi.com/article/10.3390/jcm15218258/s1, Supplementary File S1: PRISMA Check list; Supplementary File S2: Detailed Review Methodology; Supplementary Table S1: Complete Database-Specific Search Strategies; Supplementary Table S2: Data-Extraction Framework.

## Abbreviations

The following abbreviations are used in this manuscript:

CINAHL	Cumulative Index to Nursing and Allied Health Literature
ED	Emergency department
EM	Emergency Medicine
ER	Emergency Room
JBI	Joanna Briggs Institute
MMAT	Mixed Methods Appraisal Tool
n	Sample size
PIO	Population, Interest, Outcome
PRISMA	Preferred Reporting Items for Systematic Reviews and Meta-Analyses
RN	Registered Nurse
USA	The United States of America
