# Supplementary material for: Distraction-Related Phenomena in Emergency Department Work: A Systematic Literature Review from a Cognitive Ergonomics Perspective
Source: J Clin Med. 2026 Oct 23;15(21):8258. doi: 10.3390/jcm15218258 (PMC13764191; doi:10.3390/jcm15218258)
Supplement: Supplementary file 1 [file jcm-15-08258-s001.zip › Supplementary File S2.pdf]

Supplementary file S2

Detailed Review Methodology

Associated manuscript: Distraction-Related Phenomena in Emergency Department Work: A Systematic Literature Review from a Cognitive Ergonomics Perspective

1.1. Status and purpose of this document

This supplementary document provides an expanded account of the methodological procedures reported for the systematic literature review. The review was not prospectively registered, and no standalone formal review protocol was prepared. This document has been compiled retrospectively for reporting transparency; it is not presented as an original, prospectively dated protocol and cannot independently verify the timing of methodological decisions. The research question, eligibility criteria, information sources, and general methodological approach were developed during the initial planning phase. Specific coding categories and interpretative relationships were developed during the inductive synthesis.

1.2. Review question and scope

The review was structured using the Population–Interest–Outcome (PIO) framework. Population: healthcare professionals working in emergency departments (EDs). Interest: distraction-related phenomena, including interruptions, environmental and technology-related distractions, competing clinical demands, workflow disruptions, and related work processes. Outcomes: cognitive, workflow-related, emotional, professional well-being, and patient-related consequences.

Review question: "What distraction-related phenomena affect healthcare professionals in emergency departments, what consequences do they have, and under which organisational and sociotechnical conditions may interruptions be disruptive or beneficial?"

1.3. Eligibility criteria

The eligibility criteria reported in the manuscript are reproduced below. The absence of a dated standalone protocol means that their exact historical wording cannot be independently established from this supplementary document.

Domain	Included	Excluded
**Population**	Healthcare professionals working in EDs	Healthcare professionals not working in emergency departments
**Phenomenon of interest**	Identifiable interruptions, distractions, competing	Studies addressing only workload, stress, emotions,

	demands, workflow disruptions, or related multitasking/task-switching processes	situation awareness, or performance without an identifiable relevant phenomenon
**Outcomes**	Cognitive functioning, workflow/task performance, emotional responses, staff well-being, patient experience or safety	No outcome relevant to the review question
**Study type**	Full-text, peer-reviewed primary quantitative, qualitative, or mixed-methods studies	Review articles as units of analysis; theses, dissertations, grey literature, conference outputs, preprints, commentaries, opinions, unpublished reports
**Language**	English	Non-English publications

Eligibility was assessed in relation to the functional role of a construct in each study, rather than its label alone. For ambiguous studies, reviewers considered the stated aim, operational definitions, exposure–outcome relationship, and analytical framing. Reviews could be used for reference-list screening but were not themselves units of synthesis.

1.4. Information sources and search approach

The review searched PubMed, CINAHL Ultimate, Web of Science, and ScienceDirect. Supplementary searching involved Google Scholar (the first five pages of results screened by relevance) and manual screening of reference lists from eligible studies and relevant reviews. The final search was conducted on 15 March 2026. No publication-year restriction was applied; eligible reports were restricted to English-language publications.

Main search expression reported in the manuscript:

("ER" OR "emergency room" OR "emergency department" OR "ED") AND (distraction* OR "distracting event*" OR noise* OR interference* OR disturb* OR interruption* OR "work interruption*" OR "cognitive load*" OR "alarm*") AND ("stress level*" OR "patient care" OR "mental stress") AND ("healthcare worker*" OR nurse* OR "medical staff").

ScienceDirect adapted expression: ("emergency department" OR "emergency room") AND (distraction OR noise OR "work interruption") AND ("healthcare worker" OR nurse) AND (stress OR "patient safety"). Database-specific full search strategies should be consulted in Supplementary Table S2 of the manuscript, if submitted separately.

1.5. Study selection and data extraction

Three reviewers (M.M., P.K., and J.M.) independently screened titles/abstracts and potentially eligible full texts. Disagreements were resolved through discussion and consensus, with B.K. and Z.F. consulted where necessary. Full-text exclusion reasons were documented. Inter-rater agreement was not formally quantified.

Two reviewers independently extracted the complete predefined dataset from each included study using a standardised form; extracted records were compared and discrepancies resolved by consensus. The form was not formally piloted. Extracted fields included authors, publication year, country, design, setting, participants, sample size, investigated distraction phenomena, outcome measures, and findings relevant to the review question.

1.6. Methodological critical appraisal

Two reviewers (M.M. and P.K.) independently conducted design-specific appraisal. The Joanna Briggs Institute (JBI) analytical cross-sectional checklist was used for quantitative cross-sectional/descriptive observational studies; the JBI cohort checklist for the prospective cohort study; the JBI qualitative checklist for qualitative studies; and the Mixed Methods Appraisal Tool (MMAT, 2018) for mixed-methods studies. Disagreements were resolved by consensus, with additional reviewers consulted when necessary.

Appraisal results were interpreted by methodological domain rather than combined into numerical quality scores. Studies were not excluded solely on appraisal findings. Particular attention was paid to confounding, measurement validity and reliability, follow-up where applicable, qualitative reflexivity, and mixed-methods integration.

1.7. Synthesis strategy and analytical procedure

Because the studies differed substantially in design, measured constructs, and outcomes, statistical pooling was not undertaken. The reported synthesis used inductive content analysis following the approach described by by Elo and Kyngäs, comprising preparation, organisation, and reporting phases. Findings were coded at the level reported in the primary studies; quantitative results (frequencies, associations, and task-performance outcomes) and qualitative accounts (experiences, perceptions, and interpretations) were considered together while preserving their evidential differences.

In preparation, extracted findings were read repeatedly to identify meaningful units. In organisation, open codes were compared and grouped into subcategories, broader generic categories, and an overarching category. In reporting, categories were narratively synthesised and linked to contributing studies. Initial coding and categorisation were performed by P.K.; Z.F. and M.M. reviewed the developing structure, and disagreements were resolved through discussion.

Measured findings were distinguished from interpretations offered by primary-study authors and from higher-level interpretations generated by the review team. Observational associations were not treated as causal effects, and cognitive mechanisms or patient-safety consequences not directly measured were presented as interpretations.

1.8. Initial methodological decisions versus emergent analytical elements

Initial planning / reported methods	Developed or refined during analysis
**Research question, ED population, phenomenon of interest, outcome domains, and general eligibility framework**	Specific open codes and the content of subcategories and generic categories
**Information sources, selection and extraction procedures as described in Methods**	Grouping and interpretation of recurring patterns across heterogeneous studies
**General decision to synthesise heterogeneous evidence qualitatively; reported inductive content-analysis procedure**	Detailed conceptual relationships between interruption context, cognitive demands, clinician adaptation, workflow, and patient-safety implications
**Cognitive ergonomics as the overarching perspective for the review**	Application of cognitive ergonomics to interpret the categories after inductive derivation, rather than as fixed a priori coding categories

This table distinguishes the methodological approach described for the review from analytic products that necessarily arose through inductive interpretation. It is not a substitute for a prospectively archived protocol. Where a specific decision cannot be verified as having preceded screening, the manuscript should avoid describing it as independently verifiable prespecification.

1.9. Reporting limitations and transparency

The lack of prospective registration and a standalone, publicly accessible protocol limits independent verification of the chronology of methodological decisions and of potential deviations from the initial plan. This supplementary description provides additional transparency about the reported methods but cannot remove that limitation. The English-language restriction, exclusion of grey literature, and heterogeneous evidence base may also affect comprehensiveness and interpretation.
