# Supplementary material for: Distraction-Related Phenomena in Emergency Department Work: A Systematic Literature Review from a Cognitive Ergonomics Perspective
Source: J Clin Med. 2026 Oct 23;15(21):8258. doi: 10.3390/jcm15218258 (PMC13764191; doi:10.3390/jcm15218258)
Supplement: Supplementary file 1 [file jcm-15-08258-s001.zip › Supplementary Table S1.pdf]

Supplementary Table S1. Complete database-specific search strategies.

The literature search was conducted in PubMed, CINAHL Ultimate, Web of Science, and ScienceDirect and was supplemented by Google Scholar and manual reference-list screening. The final search was conducted on 15 March 2026. No publication-year restriction was applied, and only studies published in English were considered eligible. Search terms were adapted to the indexing systems and technical requirements of the individual databases while maintaining the conceptual domains of the review.

Database / source	Complete search strategy	Limits / additional information
**PubMed**	("ER" OR "emergency room" OR "emergency department" OR "ED") AND (distraction* OR "distracting event*" OR noise* OR interference* OR disturb* OR interruption* OR "work interruption*" OR "cognitive load*" OR "alarm*") AND ("stress level*" OR "patient care" OR "mental stress") AND ("healthcare worker*" OR nurse* OR "medical staff")	English language; no publication-year restriction
**CINAHL Ultimate**	("ER" OR "emergency room" OR "emergency department" OR "ED") AND (distraction* OR "distracting event*" OR noise* OR interference* OR disturb* OR interruption* OR "work interruption*" OR "cognitive load*" OR "alarm*") AND ("stress level*" OR "patient care" OR "mental stress") AND ("healthcare worker*" OR nurse* OR "medical staff")	English language; no publication-year restriction
**Web of Science**	("ER" OR "emergency room" OR "emergency department" OR "ED") AND (distraction* OR "distracting event*" OR noise* OR interference* OR disturb* OR interruption* OR "work interruption*" OR "cognitive load*" OR "alarm*") AND ("stress level*" OR "patient care" OR "mental stress") AND ("healthcare worker*" OR nurse* OR "medical staff")	English language; no publication-year restriction
**ScienceDirect**	("emergency department" OR "emergency room") AND (distraction OR noise OR "work interruption") AND ("healthcare worker" OR nurse) AND (stress OR "patient safety")	Simplified search string because of limitations in Boolean operators and keyword combinations; English language; no publication-year restriction
**Google Scholar**	("ER" OR "emergency room" OR "emergency department" OR "ED") AND (distraction* OR "distracting event*" OR noise* OR interference* OR disturb* OR interruption* OR "work interruption*" OR "cognitive load*" OR "alarm*") AND	Supplementary search; results screened in order of relevance; screening limited to the first five pages (50 records)

	("stress level*" OR "patient care" OR "mental stress") AND ("healthcare worker*" OR nurse* OR "medical staff")	
**Reference-list screening**	Reference lists of eligible articles and relevant review papers were manually screened to identify additional potentially eligible primary studies.	Backward citation searching
