# Supplementary material for: Distraction-Related Phenomena in Emergency Department Work: A Systematic Literature Review from a Cognitive Ergonomics Perspective
Source: J Clin Med. 2026 Oct 23;15(21):8258. doi: 10.3390/jcm15218258 (PMC13764191; doi:10.3390/jcm15218258)
Supplement: Supplementary file 1 [file jcm-15-08258-s001.zip › Supplementary Table S2.pdf]

Supplementary Table S2. Data-extraction framework

Data-extraction domain	Information extracted
Study identification	Author(s), year of publication
Geographical context	Country in which the study was conducted
Study characteristics	Study design and study setting
Participant characteristics	Healthcare professional group(s), participant characteristics, and sample size
Distraction-related phenomena	Type and source of distraction-related phenomena investigated, including interruptions, environmental or technology-related distractions, competing demands, multitasking, task switching, or other relevant phenomena
Outcome measures	Cognitive, emotional, workflow-related, performance-related, organisational, and patient-safety outcomes reported in relation to distraction-related phenomena
Main findings	Principal findings relevant to the review question, including reported consequences and, where applicable, context-dependent or potentially functional effects of interruptions
Review synthesis	Information relevant to the categorisation and synthesis of findings and their subsequent interpretation from a cognitive ergonomics perspective.

Extraction procedure: Data were independently extracted from all included studies by two reviewers using the same standardised data-extraction framework. The complete predefined dataset was extracted independently by both reviewers. The two extracted datasets were subsequently compared, and discrepancies were resolved through discussion and consensus.
